# Thrush nightingales adjust the peak frequency and structure of their songs in response to different types of experimental noise

**DOI:** 10.1038/s41598-025-85991-3

**Published:** 2025-01-21

**Authors:** Michał Budka

**Affiliations:** Department of Behavioural Ecology, Adam Mickiewicz University in Poznań, Uniwersytetu Poznańskiego 6, Poznan, 61614 Poland

**Keywords:** Behavioural ecology, Evolutionary ecology, Animal behaviour

## Abstract

Animals employ various strategies to minimize the overlap of their vocalizations with other sounds, thereby enhancing the effectiveness of their communication. However, little attention has been given to experimentally examining how the structure of the acoustic signal changes in response to various kinds of disturbances in the soundscape. In this study, I experimentally investigated whether male thrush nightingales (*Luscinia luscinia*) adjust their singing rate, song frequency, and song type in response to different types of artificial sounds. While males did not reduce the intensity of their singing in noise, they did adjust the peak frequency of their songs—raising it in the presence of low-frequency noise and lowering it in response to high-frequency noise. These bidirectional changes were associated with selective use of phrases from their repertoire. The proportion of low- to high-frequency phrases was significantly reduced under low-frequency noise and increased under high-frequency noise. The results suggest that disturbing sounds can modify the peak frequency and structure of songbird vocalizations, at least in the short term. Birds may enhance the signal-to-noise ratio by selecting phrases from their repertoire that are less affected by noise. For species with a large repertoire, new sounds transmitted within a species-specific frequency range may play an important role in the process of cultural song transmission.

**Supplementary Information:**

The online version contains supplementary material available at 10.1038/s41598-025-85991-3.

## Introduction

The acoustic niche hypothesis assumes that acoustic space – the time and frequency spectrum available for acoustic signals – is a limited resource for which vocalizing animals compete^1^. Studies on anthropogenic noise has strongly supported this hypothesis, demonstrating that birds adjust their vocal activity or modify the frequency and amplitude of their songs in response to noise^2–4^. However, anthropogenic noise is characterized by high intensity, continuous, and concentration in low frequencies, which primarily affects species that also produce low-frequency vocalizations. As a result, the only feasible spectral adaptations to mitigate signal masking caused by anthropogenic noise are raising the frequency of the acoustic signal^5^ or amplifying the song’s amplitude to enhance the signal-to-noise ratio^2^.

In natural terrestrial environments, insects, frogs, crocodilians, birds, and mammals vocalize from various locations and at different times of day, producing sounds with varying amplitudes across both low and high frequency spectra. In such complex soundscape, to minimize signal interference and maximize the effectiveness of communication, animals should partition the acoustic space and avoid acoustic signal masking in both frequency and time domains. Empirical research on various animal communities provides stronger support for temporal acoustic space partitioning^6–8^ rather than for spectral partitioning^9^. However, experimental studies investigating birds’ responses to both artificial sounds and the natural vocalizations of other species have demonstrated that birds can employ both temporal and spectral strategies to avoid competing sounds in the short term^10,11^. Moreover, it remains unclear whether the observed modifications in song frequency in some bird species are due to singing the same type of syllable at a different frequencies^12^ or selecting higher- or lower-frequency syllable types from their entire repertoire, depending on the type of noise^13^. Therefore, more detailed experimental studies are needed to fully understand the mechanisms of interspecific competition for acoustic space, both in the short and long term. This is especially important during times of rapid environmental change when many species alter their abundance and distribution^14^, and the environment is increasingly polluted by anthropogenic noise^15^. Consequently, new and previously unseen communities form, where individuals compete for resources, including acoustic space, and the effects and consequences of such competition are difficult to predict.

Recent experimental studies have shown that various species may undertake different strategies to limit signal interference, such as increasing the amplitude of sound^16^ vocalizing in silence gaps^17^, vocalizing when species-specific frequency band is not occupied by other species^10^, adjusting the frequency of vocalization^12^, modifying structure and duration of song^18^ or increasing signal redundancy^11^. However, little is known about the selective use of repertoire in response to competing sounds. Theoretically, individuals may more often use song types that are less masked by competing vocalizations^13^. Such a mechanism could have a significant evolutionary impact on the cultural transmission of songs^19^. Essentially, competing sounds could disrupt the availability of various song types within a population. As a result, song types that fall outside the spectrum of competing sounds would be more frequently copied by tutees and spread more widely within the population than other types. However, such process would be very slow, as a laboratory study on great tits suggests that noise rather weakly influences changes in individual singing behaviour^20^.

In the study, I experimentally examined whether male thrush nightingales (*Luscinia luscinia*) modify singing rate, frequency and structure of their song to avoid masking by competing sounds that cover various frequency spectra. The thrush nightingale is a monogamous, long-distance migratory, territorial songbird that breeds in lowland river valleys with deciduous and mixed woodlands, small woodlots, dense thickets, and shrubs in Eastern Europe and Asia^21^. Males sing intensively at night with their loud (84–87 dB at 1 m^22^) and complex songs. The repertoire size may exceed 40 song types, containing more than 50 different song components^23^, sung across a wide frequency range. Therefore, to minimize the song masking effect, male thrush nightingales have the potential to sing the same syllable type at different frequencies or use low- and high-frequency syllables in different proportions in response to various acoustic disturbances within their territories.

In the study, I broadcast low- and high-frequency white noise to territorial males of thrush nightingales to examine whether individuals modify their singing rate, and the frequency and structure of song to reduce overlap with competing sounds. I expected that the species with complex repertoires would bidirectionally modify their use of low- and high-frequency phrases in response to various types of the noise. Therefore, in response to low-frequency noise, males should increase the proportion of high-frequency phrases, while during high-frequency noise playback, they should more often use low-frequency phrases.

## Results

I analysed a total of 21,269 phrases sung by 18 tested males of thrush nightingales. On average, each male produced 1,182 (SD = 296.5; ranging from 616 to 1,637) phrases during the experiment (30 min), and from 5 to 628 phrases per single experimental treatment (10 min; average 394, SD = 115.1). The intensity of singing, measured as the number of phrases sung by each male, significantly decreased as the night progressed and was independent of the type of treatment, treatment order, or time in the season (Table 1).

**Table 1:** Results from a GLMM examining the effects of time in the season (season), time of day (hour), treatment order (treatment order), and treatment type (low-frequency noise, high-frequency noise, control – reference category) on the number of phrases sung by 18 male thrush nightingales during the playback experiment are presented.

	Estimate	SE	z	*p*
Intercept	**6.194**	**0.2230**	**27.771**	**< 0.001**
Season	0.007	0.0076	0.975	0.330
Hour	− **0.002**	**0.0007**	− **2.537**	**0.011**
Treatment order	0.022	0.0654	0.344	0.731
Low-frequency noise	− 0.216	0.1305	− 1.655	0.098
High- frequency noise	− 0.123	0.1302	− 0.945	0.345

The data were fitted using a negative binomial distribution with a log link function. Male ID was included as a random effect.

Significant effects are highlighted in bold.

**Fig. 1:**
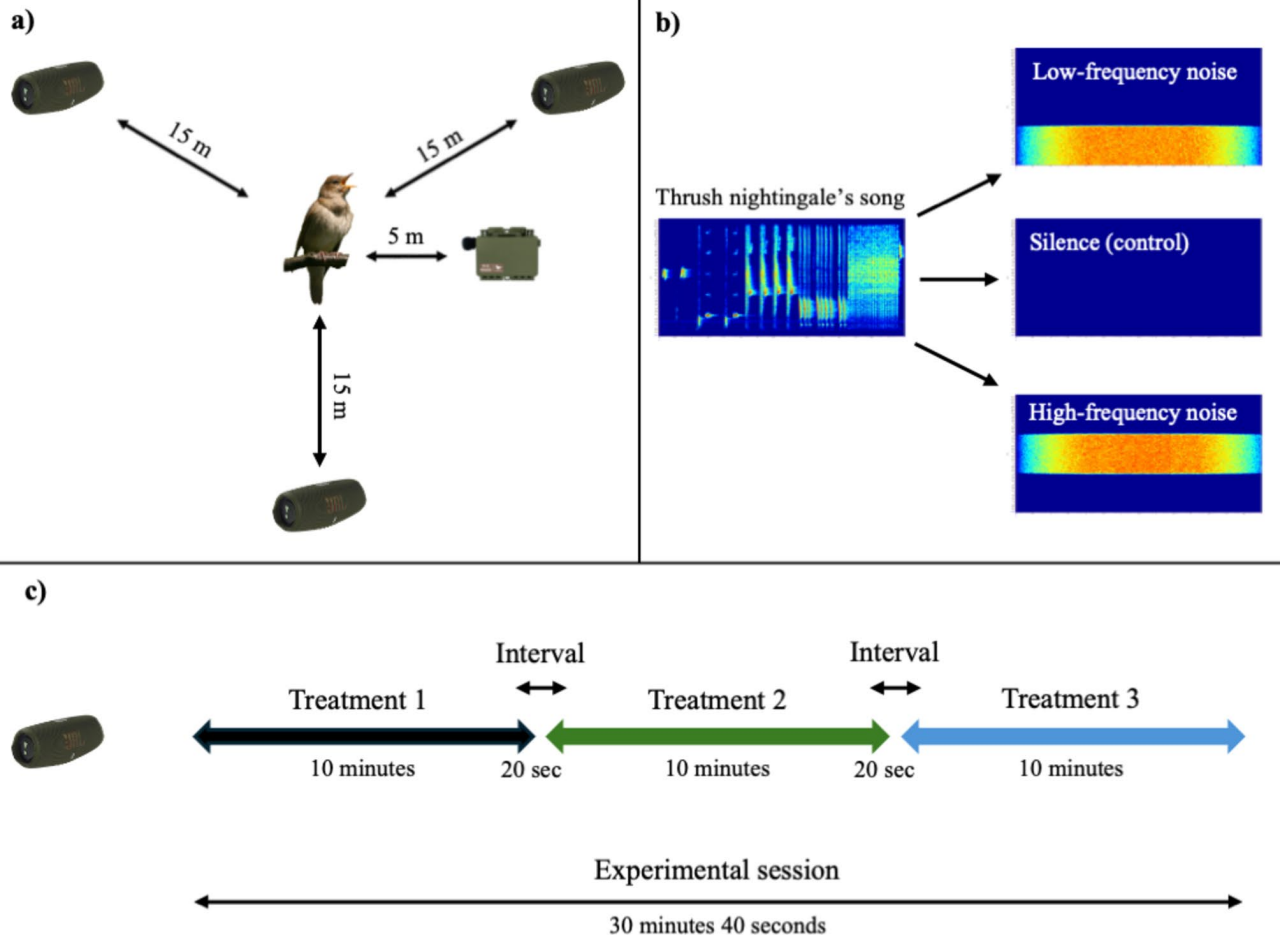
(**a**) Distribution of speakers and recorder during the experiment. (**b**) Example of a thrush nightingale’s song and the three experimental treatments: low-frequency white noise, a control with silence, and high-frequency white noise. (**c**) During the experimental session, each male was exposed to three 10-minute treatments broadcasted in random order, with 20 seconds of silence between them.

The median peak frequency of phrases sung under low-frequency noise was significantly higher, while the peak frequency of phrases sung under high-frequency noise was significantly lower than that of phrases sung during the control condition, which included silence (Fig. 1; Table 2).

**Table 2:** Results from a GLMM examining the effects of treatment order (treatment order) and treatment type (low-frequency noise, high-frequency noise, control—reference category) on the median peak frequency sung by 18 male thrush nightingales during the playback experiment are presented.

	Estimate	SE	z	*p*
Intercept	**4872.714**	**187.510**	**25.849**	**< 0.001**
Playback order	− 6.373	48.004	− 0.133	0.894
Low-frequency noise	**231.884**	**96.009**	**2.415**	**0.016**
High-frequency noise	− **237.574**	**95.563**	− **2.486**	**0.013**

The data were fitted using a Gaussian distribution with an identity link function. Male ID was included as a random effect.

Significant effects are highlighted in bold.

**Fig. 2:**
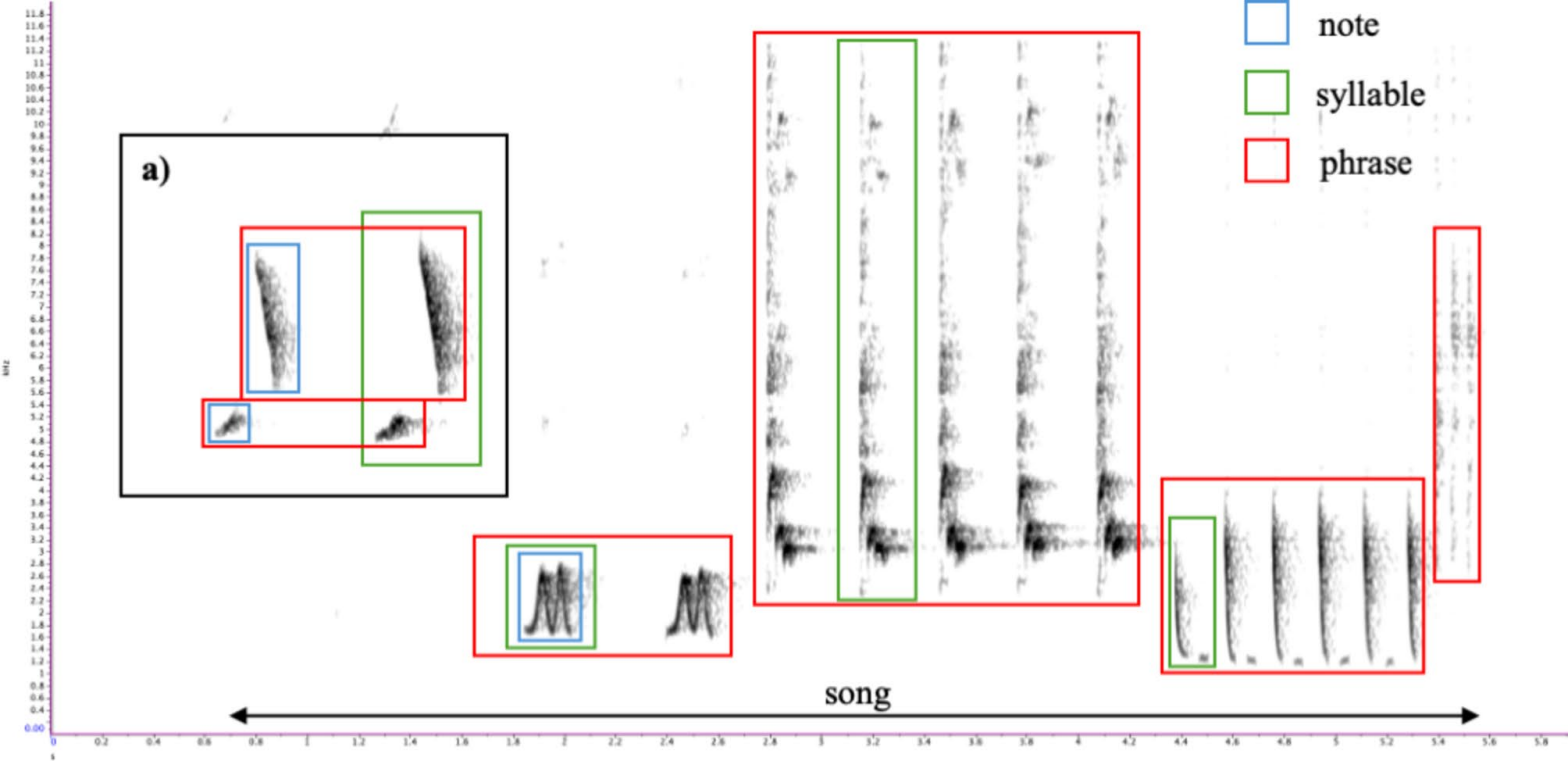
An example of a thrush nightingale’s song is shown. The song consists of several different phrases, each composed of one or more syllables. Each syllable may contain between one and three notes. In this study, the basic unit of song organization was the phrase.

The proportion of phrases with peak frequencies located below and above 4 kHz ranged from 0.25 to 3.71 (average 0.76, SD = 0.479) and was independent on the playback order. However, I found a significant effect of the type of treatment on the proportion of low- and high-frequency phrases. Compared to the control treatment (silence), males decreased the proportion of low- to high-frequency phrases during the playback of low-frequency noise (0–4 kHz) and increased the proportion during high-frequency noise (4–8 kHz) (Table 3; Fig. 2). The analysis of the number of low- and high-frequency phrases separately showed that males significantly decreased the number of low-frequency phrases during low-frequency noise compared to the control, while the change in high-frequency phrases was independent of the type of treatment noise (Table 3).

**Table 3:** Results from GLMMs analyzing the effects of treatment order and treatment type (low-frequency noise, high-frequency noise, control—reference category) on (1) the number of low-frequency phrases, (2) the number of high-frequency phrases, and (3) the proportion of low- to high-frequency phrases sung by 18 male thrush nightingales during the playback experiment are presented.

	Estimate	SE	z	*p*
	Number of low-frequency phrases			
Intercept	**5.160**	**0.1511**	**34.150**	**< 0.001**
Playback order	0.013	0.0588	0.220	0.822
Low-frequency noise	− **0.331**	**0.1212**	− **2.730**	**0.006**
High-frequency noise	− 0.037	0.1182	− 0.310	0.755

	Number of high-frequency phrases			
Intercept	**5.562**	**0.1581**	**35.18**	**< 0.001**
Playback order	− 0.012	0.0631	− 0.19	0.847
Low-frequency noise	− 0.167	0.1275	− 1.31	0.190
High-frequency noise	− 0.199	0.1257	− 1.59	0.113

	Proportion between low and high frequency phrases			
Intercept	− **0.415**	**0.0983**	− **4.22**	**< 0.001**
Playback order	0.038	0.0296	1.30	0.195
Low-frequency noise	− **0.211**	**0.0601**	− **3.51**	**< 0.001**
High-frequency noise	**0.182**	**0.0599**	**3.04**	**0.002**

Low- and high-frequency phrases are defined based on their peak frequency, being below or above 4 kHz, respectively. The data were fitted using a negative binomial distribution with a log link function (for models 1 and 2) or a Gamma distribution with a log link function. Male ID was included as a random effect.

Significant effects are highlighted in bold.

## Discussion

In natural environments, animals communicate acoustically not in silence, but within a complex soundscape composed of biological, anthropogenic, and geophysical sounds^24^. To communicate effectively, the sender of the signal must optimize their signal-to-noise ratio^2^. A straightforward way to achieve this is by vocalizing during periods of silence. Many animals have adopted such short- or long-term adjustments, vocalizing during times of day when other animals are quieter^6^ or matching their singing rate to fit into gaps between the vocalizations of other animals^11^. On the other hand, recent experimental studies have shown that noise, depending on its characteristics, can also trigger the onset of singing or increase the singing rate in some bird species^11,25^. In the closely related common nightingale (*Luscinia megarhynchos*) males significantly avoided overlapping their songs with playback songs of other bird species and began singing during the silent intervals between heterospecific songs^27^. The current experimental design did not allow the birds to short-term temporally avoid competing songs of other species, as was the case with the common nightingale^27^. Instead, it provided the opportunity to decrease singing during 10 min of continuous noise and increase it during 10 min of silence—an adjustment the males did not make. The lack of modification in singing intensity in response to noise may be attributed to two non-mutually exclusive factors: (1) the experiment was conducted in the field during the peak of seasonal (beginning of the breeding season) and daily vocal activity (at night), when males were highly motivated to singing and attract the females; and (2) the SPL of the playback was a few decibels lower than the SPL of the thrush nightingale’s song (approximately 65 dB vs. 85 dB at 1 m from a singing male), meaning the male’s song remained audible.

Only one of the 18 tested males moved away from the noise source for a few meters but did not stop singing. Considering the amplitude of thrush nightingale songs, their plasticity, the location of the speaker, and the amplitude of the experimental noise, it is possible that the males could have increased the amplitude of their songs to exceed the noise level and still be heard. Increasing the sound pressure level in noisier locations is a common strategy to improve the signal-to-noise ratio, observed in many animals, including the common nightingales^28^. Unfortunately, our experimental design did not allow us to measure this aspect of vocalizations.

Many studies have reported that increasing the frequency of songs in response to competing sounds is a common strategy. However, most of these studies compared vocalizations from locations with varying noise levels, usually anthropogenic^5,29,30^. Only a few have experimentally tested the ability of birds to shift the frequency of their vocalizations, and these have reported inconsistent results^12,18,20,31–33^. For example, great tits in the wild sang song types that were less masked by noise for a longer duration and switched to low- or high-frequency song types depending on the frequency of the noise^13^. In contrast, a laboratory study showed that males did not make any frequency or song usage adjustments when background noise conditions were altered. The results of the current study clearly showed that thrush nightingales in the wild use spectral adjustments to increase the effectiveness of their communication. Males modified the peak frequency of their songs bidirectionally, dependently on noise characteristic. They increased the peak frequency of their vocalizations under low-frequency noise and decreased it when exposed to high-frequency noise playback. The difference in the median peak frequency of phrases sung during low- and high-frequency noise levels averaged 469 Hz. This suggests limited individual adaptability to changing acoustic conditions through song frequency modification. However, from an evolutionary perspective, such mechanism allows for improved communication efficiency within a population affected by noise over just a few generations.

Interestingly, the males thrush nightingale selectively used their repertoire to increase the signal-to-noise ratio. They significantly decreased the number of low-frequency phrases when low-frequency noise was broadcast and tended to decrease the number of high-frequency phrases when exposed to high-frequency noise (though this dependency was not statistically significant). However, when the proportion between the number of low- and high-frequency phrases was analyzed, the pattern became clear. During the control condition containing silence, that proportion oscillated around 0.74. Males decreased the proportion of low- to high-frequency phrases to 0.60 in response to low-frequency noise and increased it to 0.94 under high-frequency noise. Selective using of song types depending on the frequency spectrum of noise was also reported in black-capped chickadees (*Poecile atricapillus*)^32^ or great tits (*Parus major*) in nature^13^ but not in the wild^20^. This suggests that songbirds with a large repertoire may use it selectively to improve the signal-to-noise ratio and enhance the effectiveness of their communication. However, more detailed studies are needed to understand why, in some cases, species apply this adjustment while in others they do not. Importantly, the current study showed that shifting the frequency of the song does not only involve increasing it; it can also involve lowering the frequency of the song when the competing sound is in the higher frequency spectrum. From the perspective of cultural transmission, the selective use of a songbird’s repertoire in response to competing sounds may be an important factor driving the evolution of acoustic signals^19,31^. This seems particularly important in times when many species are changing their ranges and population sizes, leading to the formation of entirely new communities with species competing for resources, including acoustic space. The short-term modifications in song frequency and song structure observed here, due to their limited range, do not allow individuals to significantly increase the efficiency of acoustic communication in different types of noise. At the same time, they demonstrate an evolutionary mechanism that, over a few generations, can substantially improve the communication efficiency in a population inhabiting a noise-polluted environment with both low and high frequencies.

## Methods

### Experiment design

The study was conducted in Bagno Calowanie, located 30 km southeast of Warsaw, Poland (central coordinates: 52.009523° N, 21.353307° E). Bagno Całowanie is a large complex of peat bogs extensively used for agriculture, with many forests, wooded areas, and bushes along drainage ditches and small wetlands. Approximately 5,000 hectares are protected under the Natura 2000 Habitat Directive (Ostoja Bagno Całowanie PLH140001) and the Birds Directive (Bagno Całowanie PLB140011). The thrush nightingale was a common breeding bird species in the study area, inhabiting forest edges, woodlands, and bushes.

**Fig. 3:**
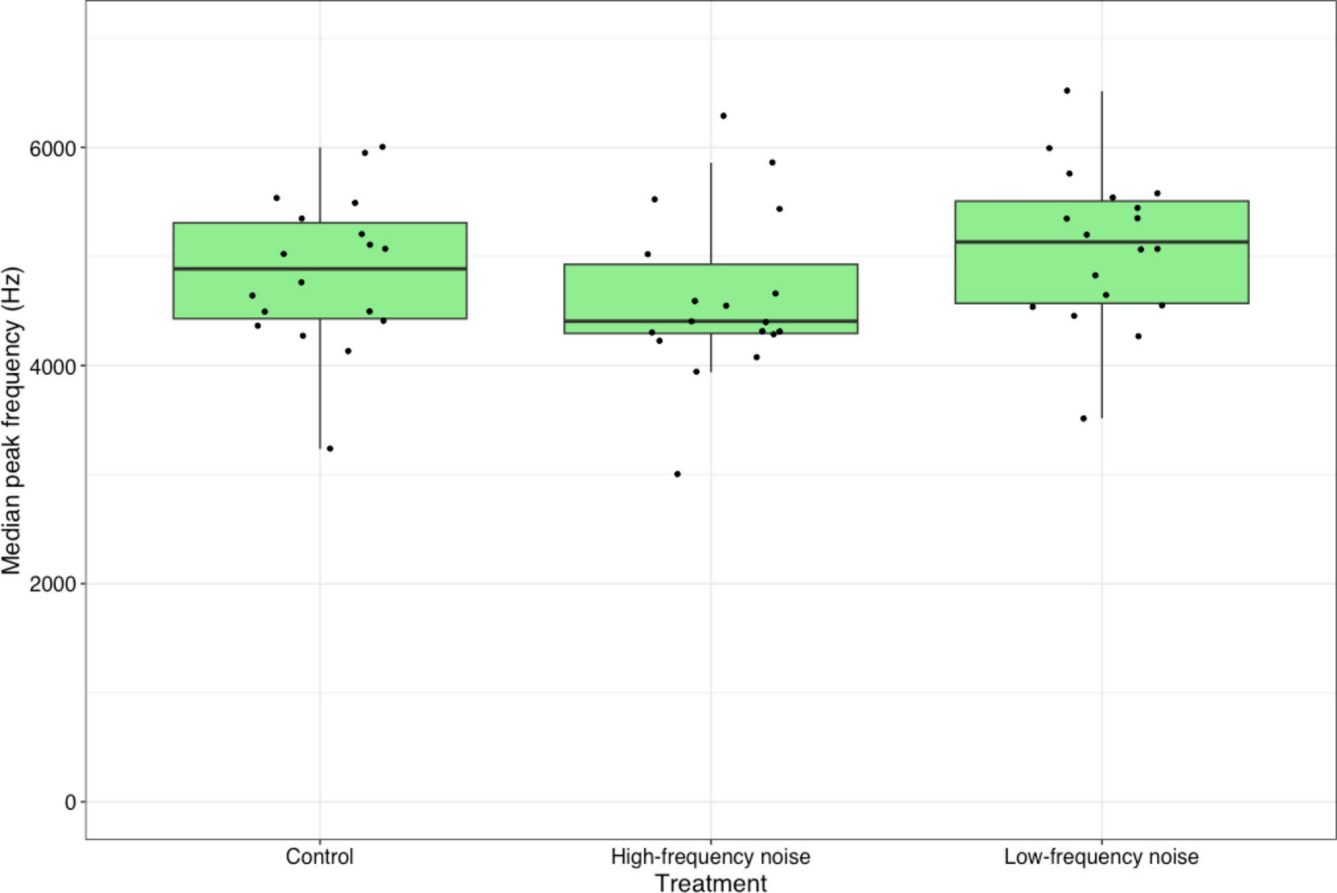
Median peak frequency of phrases sung by 18 males across three experimental treatments: high-frequency white noise (4-8 kHz), low-frequency noise (0-4 kHz), and a control conditions with silence. The graph displays the median values, interquartile range, and both minimum and maximum values.

I conducted the study from May 4th to June 6th, 2024. One to four days before the experiment, I visited the study area at night to locate the territories of the thrush nightingales and determine the exact singing location of each male. For the experiment, I selected only those males that sang more than 200 m from each other, to minimise the effect of neighbouring individuals on the behaviour of tested male. The playback experiments were conducted at night, from 9:31 PM to 2:26 AM, when males sing almost continuously. I placed a Song Meter Mini approximately 5 m from the singing male to record the response of the tested individual (wave file, 48 kHz/16 bit, gain = 6 dB, recording mode = highest quality). Three JBL Charge 5 speakers were placed approximately 15 m away from the tested bird, forming a triangle. The speakers were mounted on poles 1.5 m above the ground (Fig. 3). I started the playback approximately 5 min after placing the recorder and speakers, always when the male was singing.

Each male was exposed to three experimental treatments: (1) low-frequency noise (0–4 kHz), (2) high-frequency noise (4–8 kHz), and (3) silence as a control. The frequency range of the noise was estimated based on the measurements of thrush nightingale recordings from Poland. The idea was to set a threshold of noise to cover around half of the lowest and highest types of phrases sung by each male. The measurements suggested that, in most of the males, approximately the same number of phrases is located below and above the 4 kHz frequency. In the experiment I used white noise generated in Avisoft-SASLab Pro software (v. 5.2.09, Avisoft GmbH, Berlin, Germany). The SPL of the playback was set to 90 dB at 1 m from the speaker (measured with a sound level metre, Unit-T UT352). Considering the distance between the singing birds and the speakers (approximately 15 m) and the loss of sound intensity with distance (a decrease of 6 dB with each doubling of the distance), I estimate that the SPL of the artificial noise generated by the speakers was approximately 65 dB at the song post of the singing male. This value was lower than the SPL reported for singing thrush nightingales (84–87 dB at 1 m^22^). Each male was exposed to all three treatments in random order during a single experimental session. The duration of each treatment was 10 min, with a 20-second interval between treatments. Thus, the entire experimental procedure for a single male lasted 30 min and 40 s (Fig. 1).

### Acoustic analysis

**Fig. 4:**
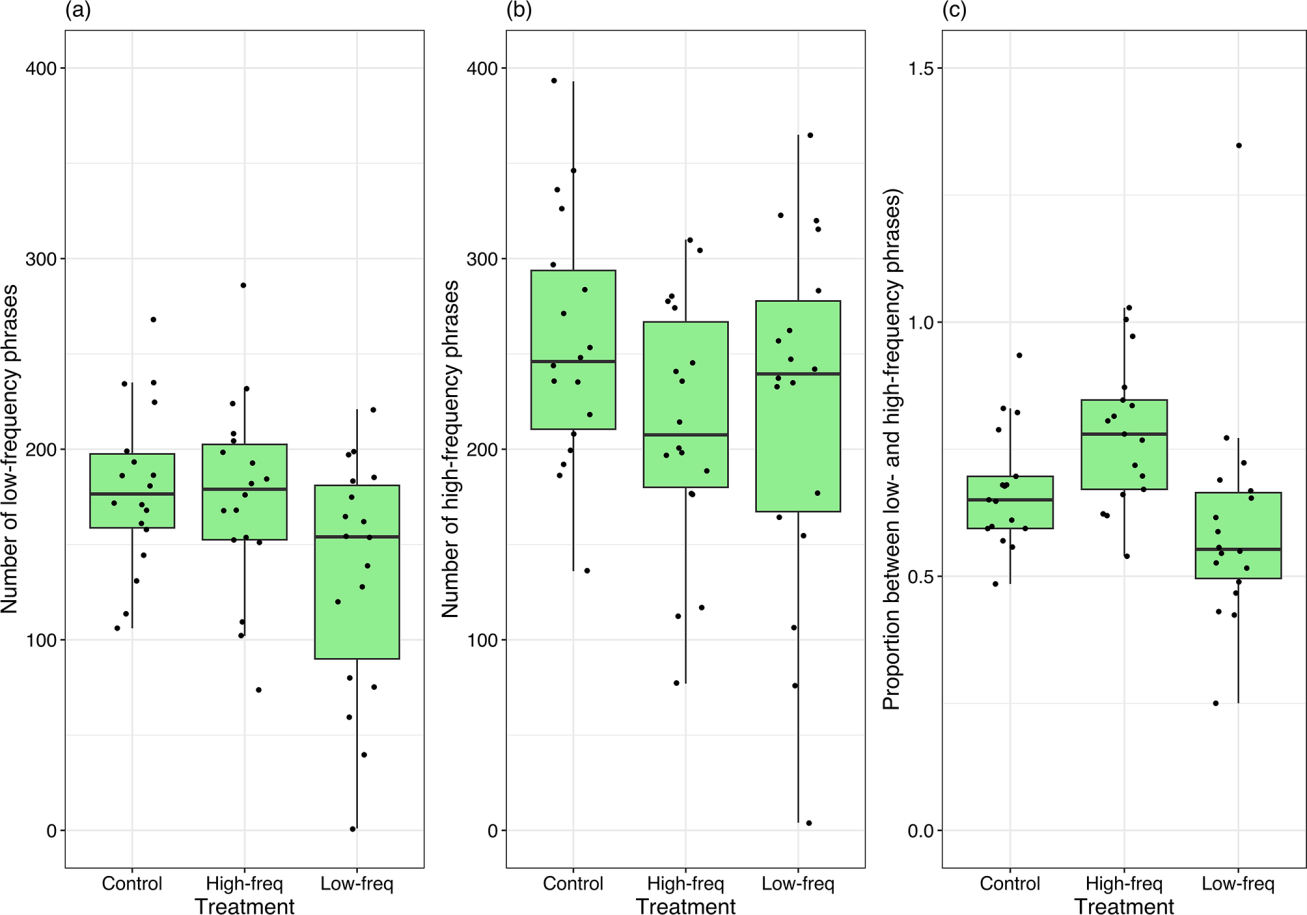
Graph shows (**a**) the number of low-frequency phrases (peak frequency < 4 kHz), (**b**) the number of high-frequency phrases (peak frequency > 4 kHz), and (**c**) the proportion between low- and high-frequency phrases sung by 18 tested thrush nightingale’s males during three experimental treatments: high-frequency white noise (4-8 kHz), low-frequency noise (0-4 kHz), and a control conditions with silence. The graph displays the median values, interquartile range, and both minimum and maximum values.

Analysing the vocal response of thrush nightingales to the playback, I focused on the phrase as the basic unit of song organization. A phrase is a sequence of the same syllable type repeated once or several times. The syllable may contain one or two notes (Fig. 4). I manually marked each phrase recorded during the 10-minute experimental treatments and automatically measured the peak frequency of each phrase. In cases where a syllable contained two notes of similar energy but significantly different frequencies (i.e., low- and high-frequency notes), I split the phrase into two parts containing only low- and high-frequency notes (Fig. 4). The experimental design made it impossible to reliably measure other parameters of songs under various levels of noise, such as phrase duration, minimum frequency, or type of syllable. All measurements were conducted automatically using Raven Pro 1.6.5 software (window type = Hann, FFT = 1024, overlap 87%, peak frequency function).

### Statistics

In the first step, I examined whether the number of phrases sung by males depended on the type of treatment, treatment order, time in the season, or time of day. I conducted a GLMM with the number of phrases as the dependent variable, type of treatment, treatment order, time in the season, and time of day as predictors, with male ID as a random effect. Data were fitted using a negative binomial distribution and a log-link function.

To detect whether males modify the peak frequency of their song in response to the experimental noise, I calculated median peak frequency for phrases produced by each male in each treatment. To minimise the effect of outliers, before the calculation I removed 1% of the lowest and 1% of the highest values from the dataset. The presence of outlier values was due to the automatic measurement of sound parameters. Within the selection containing the thrush nightingale’s phrase, there could have been sounds that were not part of the nightingale’s song (e.g., crackles, sounds from other species) that were measured automatically. By removing the outliers, these erroneous measurements were eliminated. I conducted a GLMM in which the dependent variable was the median peak frequency during each treatment. The factors included type of experimental treatment and treatments order. Male ID was applied as a random effect. The data were fitted using a Gaussian distribution and identity link function.

In the last step, for each experimental treatment I calculated the number of phrases which peak frequency was located below and above the 4 kHz. After that I conducted three separate GLMMs considering (1) number of phrases with the peak frequency lower than 4 kHz, (2) number of phrases with the peak frequency higher than 4 kHz, and (3) the proportion between the number of low- and high-frequency phrases (i.e. characterised by peak frequency located below and above 4 kHz, respectively). As predictors I applied type of treatment and treatment order. The male ID was applied as a random effect. Data were fitted by negative binomial distribution with a log-link function (models 1 and 2) or by gamma distribution with log link function (model 3). The analyses were conducted using glmTMB^34^, DHARMa^35^, vegan^36^ and ggplot2^37^ packages in R 4.3.3. Raw data can be found in the Supplementary Materials (Table S1).

## Electronic supplementary material

Below is the link to the electronic supplementary material.

Supplementary Material 1

## Data Availability

Data is provided within the manuscript and supplementary information files.
